# Do Individuals With Autism Spectrum Disorders Help Other People With Autism Spectrum Disorders? An Investigation of Empathy and Helping Motivation in Adults With Autism Spectrum Disorder

**DOI:** 10.3389/fpsyt.2019.00376

**Published:** 2019-06-04

**Authors:** Hidetsugu Komeda, Hirotaka Kosaka, Toru Fujioka, Minyoung Jung, Hidehiko Okazawa

**Affiliations:** ^1^Department of Education, College of Education, Psychology and Human Studies, Aoyama Gakuin University, Tokyo, Japan; ^2^Department of Neuropsychiatry, Faculty of Medical Sciences, University of Fukui, Fukui, Japan; ^3^Research Center for Child Mental Development, University of Fukui, Fukui, Japan; ^4^Biomedical Imaging Research Center, Division of Medical Imaging, University of Fukui, Fukui, Japan

**Keywords:** empathy, helping, autism spectrum disorders, social cognition, alexithymia, social skill

## Abstract

Individuals with autism spectrum disorder (ASD) often lack cognitive empathy, so they experience difficulty in empathizing with others. Although deficits in social abilities, such as empathy, have been demonstrated in previous studies, most stimuli used in previous studies were developed for typically developing (TD) individuals. Previous studies have demonstrated that adults with and without ASD display empathetic responses toward similar others. Adults with ASD (*n* = 22, 7 women and 15 men, mean age = 26.8 years) and intelligence- and age-matched TD adults (*n* = 20, 8 women and 12 men, mean age = 24.0 years) participated in the study. They were instructed to read 24 stories (12 stories featured protagonists with characteristics of ASD, and the other 12 featured TD protagonists) and respond to the following questions: “How did the protagonist feel?” and “Would you help if the protagonist were in trouble?” After controlling for alexithymia and AQ based on multiple regression analyses, individuals with ASD empathize with other people who have ASD and are motivated to help other people with ASD. Additionally, social skills and attention to detail were associated with decreased helping motivation for story characters with ASD. Social skills among AQ subscales (social skills, attention switching, attention to detail, communication, and imagination) were the most potent predictor of decreased helping motivation. These findings suggest that the reason why individuals with ASD are considered to have limited cognitive empathy and helping motivation could be related to alexithymia and the lack of social skills and attention to detail, which are related to atypical perception.

## Introduction

Autism spectrum disorder (ASD) is characterized by difficulties with reciprocal social interaction, atypical communication, repetitive behaviors, and narrow interests (1). Empathy plays a crucial role in communication because it enables individuals to understand another’s feelings and to use judgment to assess others’ actions (2, 3). It is known that empathy does not always lead to helping behaviors (4, 5). Moreover, empathy can be divided into two types: cognitive empathy, which is to identify the emotions of others, and affective empathy, which is to share or match one’s emotions with another’s (5). Furthermore, the degree of an empathetic response seemingly depends on different variables, including the similarity between people, and traits such as alexithymia.

Empathy is more likely to occur when there is a similarity between the participant and the target (6). For example, we are often more satisfied with interactions involving individuals similar to ourselves (7). People generally prefer individuals with personalities similar to their own (8, 9). People also show in-group biases toward their in-group and feel more similar to them than to members of out-groups, even when the composition of those groups is based on random assignment (10).

Individuals with ASD often lack cognitive empathy, which is the ability to attribute mental states to oneself and others and to understand that others have beliefs different from their own (11). Although deficits in social abilities, such as empathy, have been demonstrated in previous studies (12, 13), most target stimuli used in previous studies were developed for typically developing (TD) individuals. TD individuals tend to empathize with other people who are similar to themselves (14). Other studies have demonstrated that adults with and without ASD display empathetic responses toward similar others (6, 15). Using functional magnetic resonance imaging, Komeda et al. (15) examined whether individuals with ASD experience empathy toward other people with ASD. The ventromedial prefrontal cortex (vmPFC) was significantly activated in individuals with ASD in response to characters with ASD and in TD individuals in response to characters without ASD. Additionally, higher Autism-Spectrum Quotient (AQ) scores (16) in individuals with ASD were significantly correlated with greater activation in the vmPFC while reading about characters with ASD. Thus, individuals with ASD tend to empathize with others with ASD, at least on an explicit social judgment task (15). Although individuals with ASD have affective empathy toward other individuals with ASD, it is still unclear if they have cognitive empathy toward other individuals with ASD.

If an individual with ASD experiences alexithymia, it is unlikely that helping motivation will occur. This is due to dysfunction in emotional awareness, social attachment, and interpersonal relating (17). Additionally, alterations in perception may play a role in explaining deficits of social interaction in individuals with ASD. For example, higher sensory reactiveness is associated with lower social functioning (18). Alexithymia and certain aspects of sensory alterations are based on atypical interoception (19, 20). The prevalence of alexithymia in the general population is 10% (21, 22), and it is known that alexithymia frequently co-occurs in individuals with ASD (50%; 23). Given the occurrence of alexithymia in individuals with ASD, alexithymia was measured in the current study.

Previous studies that used verbal stimuli and declarative knowledge demonstrated that individuals with ASD have a preference for other individuals with ASD (24, 25). However, it remains unclear whether adults with ASD also show the motivation to help similar others as a consequence of empathetic responses. It is hypothesized that individuals with ASD are more likely to empathize with others with ASD and show a motivation to help other people with ASD, compared to TD individuals. In order to test our hypothesis, we examined cognitive empathy and helping motivation in ASD by considering the alexithymia and autistic traits.

## Method

### Participants

Japanese adults with ASD (*n* = 22, 7 women and 15 men, mean age = 26.8 years) and intelligence- and age-matched TD adults (*n* = 20, 8 women and 12 men, mean age = 24.0 years) were recruited at the Department of Neuropsychiatry at the University of Fukui Hospital, Japan. At the time of this study, the second author confirmed that none of the participants had other psychiatric disorders according to *Diagnostic and Statistical Manual of Mental Disorders* (*DSM-5*) diagnostic criteria, including depression or anxiety disorder. We followed recommended guidelines (26) and calculated our target sample size using an estimated effect size, d, of 0.45 (27), which would require a sample size of approximately 42 participants for the study to have 80% power. The effect size of a previous study (28) was used in the current study.

The second author diagnosed the participants based on the classifications in the *DSM-5* (1) and standardized criteria using the Diagnostic Interview for Social and Communication Disorders (DISCO) (29). The second author was trained in the diagnosis of ASD and certified to use the DISCO (30). He is a licensed psychiatrist and has over 20 years of clinical and research experience with individuals with ASD. The DISCO has adequate psychometric properties (31). Further, it contains items on early development and a section on activities of daily life, thereby giving the interviewer an idea of the level of functioning in several different areas, besides social functioning and communication (29).

The Autism-Spectrum Quotient (AQ) (16) was used to assess ASD symptoms in all participants (**Table 1**). AQ scores were significantly higher in the ASD (*M* = 32.8, *SD* = 6.4) than the TD group (*M* = 17.8, *SD* = 7.3). Alexithymia was measured by the Toronto Alexithymia Scale (TAS-20) (32, 33). The TAS-20 is a 20-item self-report scale that includes statements like “I have feelings that I cannot quite identify” (Difficulty Identifying Feelings), “I find it hard to describe how I feel about people” (Difficulty Describing Feelings), and “I prefer to analyze problems rather than just describe them” (Externally Oriented Thinking). Items are rated on a scale from 1 (does not describe me) to 5 (describes me very well), with scores ranging between 20 and 100, higher scores indicating more alexithymic traits. The Bermond–Vorst Alexithymia Questionnaire (BVAQ) was also used to measure the cognitive and emotional components of alexithymia (34, 35). Cognitive alexithymia consists of identifying (e.g., “When I am tense, it remains unclear from which of my feelings this comes”), analyzing (e.g., “I hardly ever consider my feelings”), and verbalizing (e.g., “I find it difficult to express my feelings verbally”), while emotional alexithymia consists of emotionalizing (e.g., “When something unexpected happens, I remain calm and unmoved”) and fantasizing (e.g., “I have few daydreams and fantasies”) (34).

**Table 1:** Mean chronological age, full-scale intelligence quotient (IQ), verbal IQ, performance IQ, total TAS-20, Difficulty Identifying Feelings, Difficulty Describing Feelings, Externally Oriented Thinking, total Bermond–Vorst Alexithymia Questionnaire (BVAQ), cognitive alexithymia, emotional alexithymia, total Autism-Spectrum Quotient (AQ) scores, social skill, attention switching, attention to detail, communication, and imagination in individuals with autism spectrum disorder (ASD) and typically developing (TD) adults.

	ASD group (*n* = 22)	TD group (*n* = 20)		*p*	
Gender			**Chi square**		
Female	7	8	0.3	*p* > .05	
Male	15	12			
			*t*		*Cohen’s d*
Age in years	26.8 (7.3)	24.0 (4.2)	1.5	*p* > .05	0.5
Full-scale IQ	108.0 (12.4)	114.4 (8.8)	−1.9	*p* > .05	0.6
Verbal IQ	111.1 (14.3)	115.7 (9.5)	−1.2	*p* > .05	0.4
Performance IQ	105.0 (13.2)	110.0 (11.7)	−1.3	*p* > .05	0.4
Total TAS-20	50.5 (23.1)	39.3 (12.3)	1.9	*p* > .05	0.6
Difficulty Identifying Feelings	31.2 (17.9)	23.9 (18.1)	1.4	*p* > .05	0.4
Difficulty Describing Feelings	19.0 (4.9)	14.4 (3.6)	3.4*	*p* < .05	1.1
Externally Oriented Thinking	21.4 (3.3)	18.1 (3.1)	3.3*	*p* < .05	1.0
Total BVAQ	90.6 (45.9)	85.6 (37.1)	0.4	*p* > .05	0.1
Cognitive alexithymia	73.6 (12.3)	64.8 (8.8)	2.7*	*p* < .05	0.8
Emotional alexithymia	58.8 (32.0)	63.6 (41.0)	−0.4	*p* > .05	0.1
Total AQ	32.8 (6.4)	17.8 (7.3)	6.8*	*p* < .05	2.2
Social skill	8.4 (1.7)	3.5 (2.7)	6.8*	*p* < .05	2.2
Attention switching	7.1 (1.9)	4.2 (2.2)	4.3*	*p* < .05	1.4
Attention to detail	5.3 (2.5)	4.3 (2.3)	1.3	*p* > .05	0.4
Communication	6.5 (2.2)	3.0 (2.2)	5.0*	*p* < .05	1.6
Imagination	5.6 (1.9)	2.9 (1.6)	4.8*	*p* < .05	1.5

Means (SDs) are presented.

*p < .05. Results based on two-sample t-tests.

Cognitive alexithymia consists of identifying, analyzing, and verbalizing, while emotional alexithymia consists of emotionalizing and fantasizing, based on Vorst and Bermond (35).

**Table 1** shows the mean chronological age; full-scale intelligence quotient (IQ); verbal IQ; performance IQ; total TAS-20; means of the TAS-20 Difficulty Identifying Feelings items, TAS-20 Difficulty Describing Feelings items, and TAS-20 Externally Oriented Thinking items; total BVAQ; means of the BVAQ cognitive alexithymia items and BVAQ emotional alexithymia items; total Autism-Spectrum Quotient (AQ) scores; and means of the AQ social skills items, AQ attention switching items, attention to detail items, communication items, and imagination items in adult individuals with ASD and TD.

### Procedure

All participants completed the Wechsler Adult Intelligence Scale—Third Edition (WAIS-III) (36). Previously developed materials by the authors (14) were revised and used in the study to investigate cognitive empathy and helping motivation in ASD, and the materials were revised to include situations that required help. These materials consisted of 24 stories with 6 sentences in each narrative, such that 12 stories featured protagonists with ASD characteristics and the other 12 featured TD protagonists. Our project team, which included a certified psychiatrist, conducted a thorough analysis of the confirmation of ASD or TD in each context and each outcome. The participants of the study were not instructed to report each story character as ASD or TD, because we were only interested in investigating the implicit similarity between the participants and the story characters. Each story contained five sentences about the context (story setting and the protagonist’s characteristics) and a sixth sentence about the story outcome (**Table 2**). To avoid any confusion, we analyzed 1) the ASD context and ASD outcome stories as ASD stories and 2) TD context and TD outcome stories as TD stories^1^.

**Table 2:** Sample story involving ASD context.

Mai’s best friend deeply trusted Mai, and she was open with Mai about her important secrets.	
Mai told her roommate the secrets that her best friend had told Mai.	
Mai’s best friend got angry and asked Mai, “Why did you tell my secrets to everybody?”	
Mai replied, “You didn’t tell me that it was a secret or not to tell anyone.”	
Mai’s best friend cried and said, “Mai betrayed me.”	
ASD outcome (target sentence)	TD outcome (target sentence)
Mai did not understand why her best friend got angry.	Mai decided to apologize to her best friend after she realized how sad she was.

The stories were presented on PC laptops with the software SuperLab 5.0 (Cedrus Corporation). Participants read two stories to familiarize themselves with the reading procedure before the test session. Then, participants were instructed to read each of the stories (after reading each target sentence), which were presented one sentence at a time on a computer screen, and respond to the following questions: “How did the protagonist feel?” and “Would you help if the protagonist were in trouble?”^2^ They responded by using a seven-point scale (1: least empathy, 4: neutral, 7: greater empathy, and 1: least motivation for helping, 4: neutral, 7: greater motivation for helping). Each sentence remained on the screen until the participant pressed the space bar, which caused the next sentence to appear. The time it took for the participants to read each sentence was recorded. Participants read 26 stories including 2 practice stories, and 24 stories (experimental stories) were analyzed.

In the hierarchical multiple regression analyses, the ASD group was coded as 1, and the TD group was coded as 2 (**Tables 3** and **4**). Gender was the dummy variable (female was 0, and male was 1). The regression models included the participants’ age in years and verbal and performance IQ scores. While the gender, age, and verbal and performance IQ scores were covariates, the group (ASD or TD), alexithymia scales (TAS-20 subscales Difficulty Identifying Feelings, Difficulty Describing Feelings, and Externally Oriented Thinking, and BVAQ cognitive and emotional alexithymia scores), and AQ subscales (social skills, attention switching, attention to detail, communication, and imagination) were experimental variables. Because alexithymia is based on atypical interoception (20) and lack of emotional recognition (19), these variables were put in the second regression model. The AQ subscales were calculated as a continuous value on a spectrum of typical development to atypical development. Thus, they were put in the third regression models.

**Table 3:** Standardized regression coefficients (*beta* weights) and *R^2^* from the hierarchical regression analyses based on empathy and helping values for ASD stories.

Individual scores	Empathy		Helping motivation	
*First step*	*Beta*	*t*	*Beta*	*t*
Group (1: ASD, 2:TD)	−.33	−1.8	−.05	−0.2
Gender (0: female, 1: male)	.04	0.2	.10	0.6
Age in years	−.06	−0.3	−.12	−0.6
Verbal IQ	.08	0.4	.01	0.1
Performance IQ	−.26	−1.5	−.05	−0.2
*F*	1.62		0.20	
Adjusted* R^2^*	.08		−.12	
*Second step*	*Beta*	*t*	*Beta*	*t*
Group (1: ASD, 2:TD)	−.36	−1.9	−.27	−1.4
Gender (0: female, 1: male)	.11	0.7	.16	1.0
Age in years	−.02	−.14	−.02	−0.1
Verbal IQ	−.02	−.10	−.09	−0.5
Performance IQ	−.37	−2.0	−.36	−1.8
Difficulty Identifying Feelings	.02	0.0	−.38	−0.9
Difficulty Describing Feelings	−.10	−0.5	−.07	−0.3
Externally Oriented Thinking	−.54	−2.6*	−.55	−2.5*
Cognitive alexithymia	.36	1.9	−.10	−0.5
Emotional alexithymia	−.38	−1.0	.00	0.0
*F*	2.27*		1.84	
Adjusted *R^2^*	.26		.19	
*Third step*	*Beta*	*t*	*Beta*	*t*
Group (1: ASD, 2:TD)	−.57	−2.6*	−.68	−3.0*
Gender (0: female, 1: male)	.31	2.1	.37	2.4*
Age in years	.05	0.3	.07	0.4
Verbal IQ	.06	0.4	−.07	−0.5
Performance IQ	−.35	−2.1*	−.36	−2.0
Difficulty Identifying Feelings	−.21	−0.6	−.58	−1.5
Difficulty Describing Feelings	.01	0.1	.11	0.5
Externally Oriented Thinking	−.62	−3.3*	−.73	−3.7*
Cognitive alexithymia	.51	2.7*	.05	0.2
Emotional alexithymia	−.20	−0.6	.15	0.4
Social skill	−.65	−2.5*	−.92	−3.5*
Attention switching	.28	1.2	.20	0.8
Attention to detail	−.33	−2.3*	−.10	−0.7
Communication	.31	1.3	.22	0.9
Imagination	−.10	−0.5	−.10	−0.3
*F*	2.86*		2.53*	
Adjusted *R^2^*	.43		.38	

*p < .05, two-tailed.

Three individuals with ASD did not answer AQs. Thus, the hierarchical regression analyses were conducted for 19 individuals with ASD and 20 TD individuals.

Because VIFs (variance inflation factors) of all variables were under 9.4, multicollinearity issues were not necessary to consider.

**Table 4:** Standardized regression coefficients (beta weights) and *R* ^2^ from the hierarchical regression analyses based on empathy and helping values for TD stories.

Individual scores	Empathy		Helping motivation	
*First step*	*Beta*	*t*	*Beta*	*t*
Group (1: ASD, 2:TD)	.46	2.7*	.46	2.8*
Gender (0: female, 1: male)	−.06	−0.4	.04	0.3
Age in years	−.05	−0.3	−.10	−0.6
Verbal IQ	.18	1.1	.15	1.0
Performance IQ	−.19	−1.1	−.03	−0.2
*F*	2.53*		2.86*	
Adjusted* R^2^*	.17		.20	
*Second step*	*Beta*	*t*	*Beta*	*t*
Group (1: ASD, 2:TD)	.47	2.3*	.37	2.0
Gender (0: female, 1: male)	−.07	−0.4	.01	0.1
Age in years	.00	0.0	−.04	−0.2
Verbal IQ	.14	0.8	.07	0.5
Performance IQ	−.30	−1.4	−.24	−1.2
Difficulty Identifying Feelings	.28	0.6	.38	0.9
Difficulty Describing Feelings	−.14	−0.6	−.43	−2.0
Externally Oriented Thinking	−.21	−0.9	−.21	−1.0
Cognitive alexithymia	.08	0.4	.03	0.1
Emotional alexithymia	−.16	−0.4	−.24	−0.6
*F*	1.30		2.14	
Adjusted *R^2^*	.07		.24	
*Third step*	*Beta*	*t*	*Beta*	*T*
Group (1: ASD, 2:TD)	.45	1.6	.28	1.0
Gender (0: female, 1: male)	−.14	−0.7	.00	0.0
Age in years	−.02	−0.1	.02	0.1
Verbal IQ	.08	0.4	.02	0.1
Performance IQ	−.32	−1.4	−.27	−1.3
Difficulty Identifying Feelings	.38	0.7	.52	1.1
Difficulty Describing Feelings	−.13	−0.5	−.47	−1.9
Externally Oriented Thinking	−.24	−1.0	−.24	−1.0
Cognitive alexithymia	.02	0.1	.03	0.1
Emotional alexithymia	−.22	−0.5	−.36	−0.8
Social skill	.05	0.2	.14	0.5
Attention switching	−.25	−0.8	−.22	−0.8
Attention to detail	.29	1.5	.16	0.9
Communication	−.01	−0.0	−.09	−0.3
Imagination	.06	0.2	−.15	−0.6
*F*	1.01		1.41	
Adjusted *R^2^*	.00		.14	

*p < .05, two-tailed.

Three individuals with ASD did not answer AQs. Thus, the hierarchical regression analyses were conducted for 19 individuals with ASD and 20 TD individuals.

Because VIFs of all variables were under 9.4, multicollinearity issues were not necessary to consider.

### Data Analysis

R package anovakun_480 (anovakun version 4.8.0) (http://riseki.php.xdomain.jp/index.php?ANOVA%E5%90%9B) was used in the analysis of variance (ANOVA). IBM SPSS Statistics version 21 was used in the hierarchical multiple regression analyses.

## Results

Reading times more than 2 standard deviations above the mean of each participant were excluded from the analysis. We conducted a 2 (ASD participants/TD participants) × 2 (ASD stories/TD stories) ANOVA on reading times. Results indicated that the interaction between participants and stories was significant [*F*(1, 40) = 4.39, *p* < .05, *η_p_^2^* = .10]. TD participants read TD stories faster (2,356.1 ms) than ASD stories (2619.0 ms), which was significant [*F*(1, 19) = 6.28, *p* < .05, η*_p_^2^* = .25]. However, the ASD participants did not read ASD stories (2,516.8 ms) faster than TD (2,512.9 ms) stories [*F*(1, 21) = 0.00, *p* > .05, η*_p_^2^* = .00]. Moreover, the reading times of TD participants were shorter for similar stories than for dissimilar stories (38).

**Figure 1:**
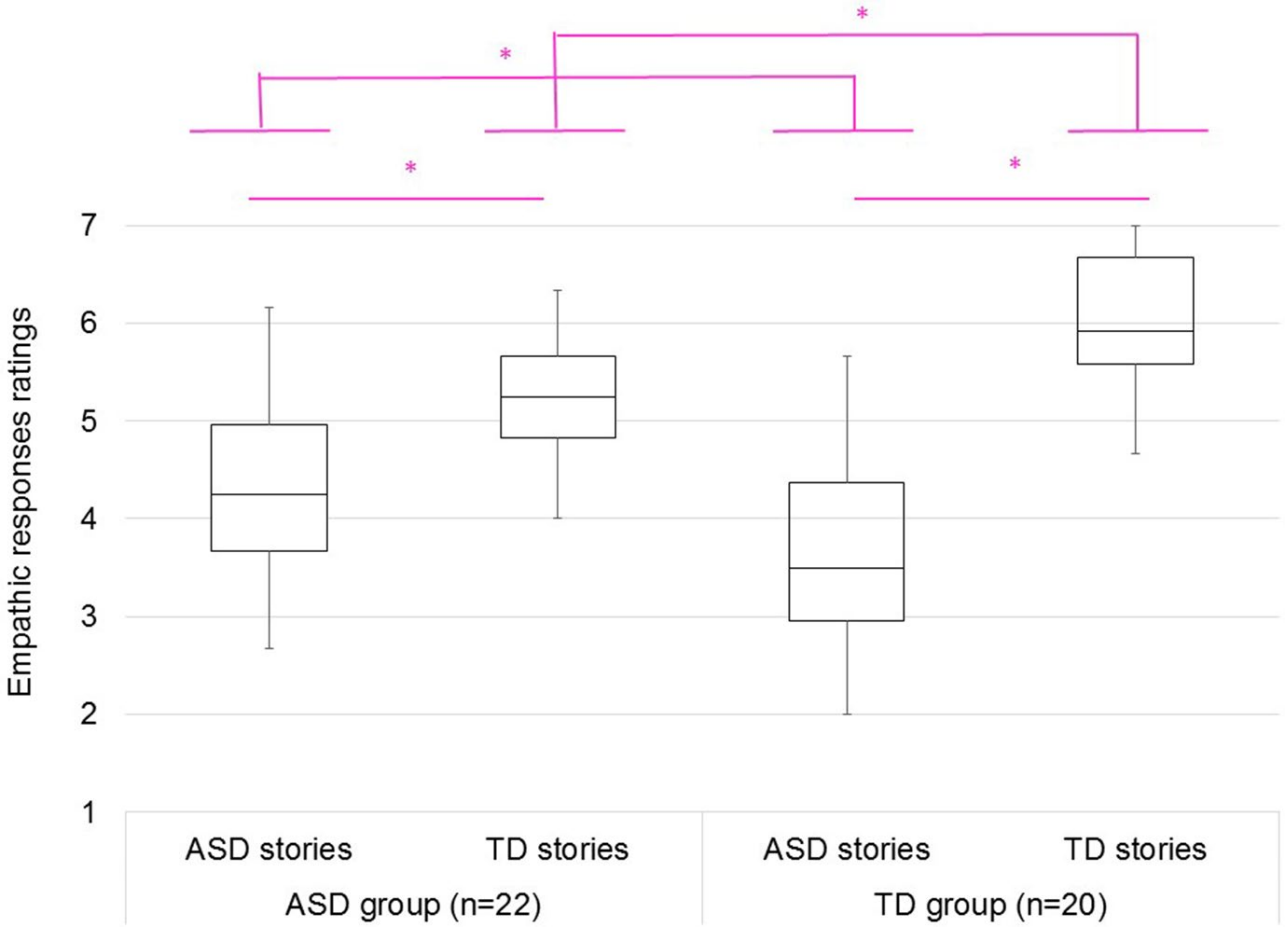
The empathic response ratings for autism spectrum disorder (ASD) and typically developing (TD) stories of ASD (left) and TD (right) groups. 1: least empathy; 4: neutral; 7: greatest empathy.

We also conducted a 2 × 2 ANOVA on empathetic response ratings (**Figure 1**). Results indicated that the interaction between participants and stories was significant [*F*(1, 40) = 14.57, *p* < .05, η*_p_^2^* = .27]. ASD participants showed greater empathetic responses in ASD stories than TD participants [*F*(1, 40) = 6.17, *p* < .05, η*_p_^2^* = .13], whereas TD participants showed greater empathetic responses in TD stories than ASD participants [*F*(1, 40) = 12.27, *p* < .05, η*_p_^2^* = .23]. Both ASD and TD participants showed greater empathetic responses in TD stories than ASD stories [*F*(1, 21) = 9.11, *p* < .05, η*_p_^2^* = .30 for ASD group; *F*(1, 19) = 63.68, *p* < .05, η*_p_^2^* = .77 for TD group].

**Figure 2:**
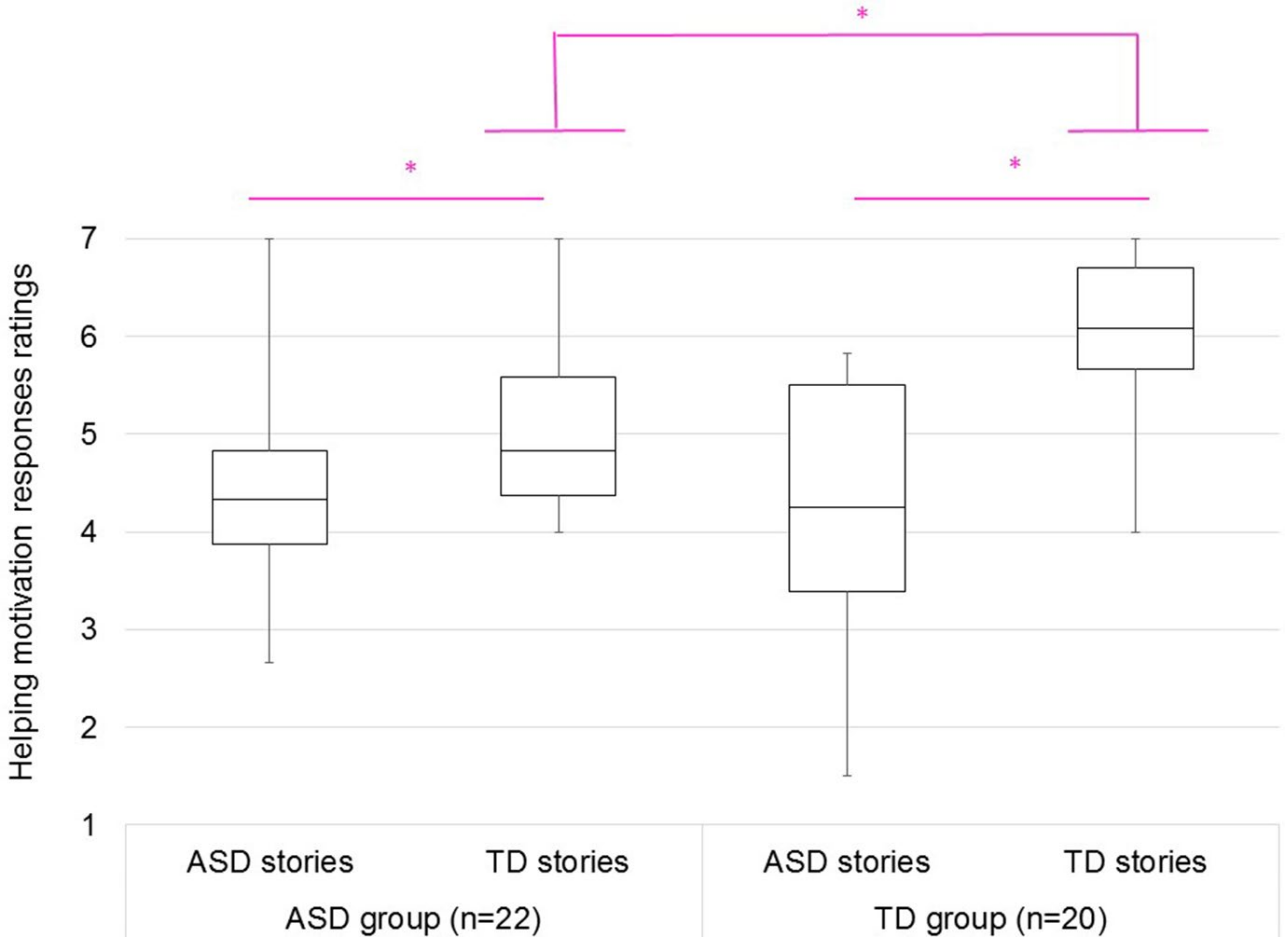
The helping motivation ratings for ASD and TD stories of ASD (left) and TD (right) groups. 1: least motivation; 4: neutral; 7: greatest motivation.

A 2 × 2 ANOVA on motivation-for-helping ratings was also conducted, which indicated that the interaction between participants and stories was significant [*F*(1, 40) = 8.40, *p* < .05, η*_p_^2^* = .17]. **Figure 2** shows that TD participants had greater motivation for helping in TD stories than ASD participants [*F*(1, 40) = 15.79, *p* < .05, η*_p_^2^* = .28], whereas ASD participants did not show increased motivation for helping in ASD stories than TD participants [*F*(1, 40) = 0.00, *p* > .05, η*_p_^2^* = .00]. However, both ASD and TD participants showed increased motivation for helping in TD stories than ASD stories [*F*(1, 21) = 8.76, *p* < .05, η*_p_^2^* = .29 for ASD group; *F*(1, 19) = 42.14, *p* < .05, η*_p_^2^* = .69 for TD group].

Hierarchical multiple regression analyses were conducted for empathetic responses and motivation-for-helping ratings to control for alexithymia characteristics (39) and AQ (16).^3^

The variables included empathic responses and helping motivation responses toward story characters in ASD stories (**Table 3**). The first regression models were not significant (*F* = 1.62 for empathy rating, *F* = 0.20 for helping rating). Although the empathy rating of the second regression model was significant (*F* = 2.27), the group variable was not significant (beta = −.36 for empathy rating). The third regression model was significant (*F* = 2.86 for empathy rating, *F* = 2.53 for helping rating). In the third model, the group (ASD: 1, TD: 2) was negatively correlated with empathy for story characters in ASD stories. Thus, the ASD group was associated with increased empathy for story characters in ASD stories. In motivation for helping, the group (ASD: 1, TD: 2) was negatively correlated with helping motivation for story characters in ASD stories. Thus, the ASD group was associated with increased helping motivation for story characters in ASD stories. In addition, cognitive alexithymia was associated with increased empathy for story characters in ASD stories. The Externally Oriented Thinking, social skills, and attention to detail variables decreased helping motivation for story characters in ASD stories.

We also analyzed the TD stories using the variables of empathic responses and helping motivation responses toward story characters in TD stories (**Table 4**). The first regression model was significant (*F* = 2.53 for empathy rating, *F* = 2.86 for helping rating). In summary, the TD group was associated with increased empathy for story characters in TD stories, and the TD group was associated with increased helping motivation for story characters in TD stories. The second and the third regression models were not significant.

## Discussion

ASD participants showed greater empathetic responses in ASD stories than TD participants, whereas TD participants showed greater empathetic responses in TD stories than ASD participants. These results suggested that the empathy for ASD story characters was higher in participants with ASD than in TD participants, whereas the empathy for TD story characters was higher in TD participants than in participants with ASD. TD participants showed greater motivation for helping in TD stories than ASD participants, whereas ASD participants did not show a greater motivation for helping in ASD stories than TD participants. These results suggested that the motivation for helping ASD story characters was similar for participants with ASD and TD participants, whereas the motivation for helping TD story characters was higher in TD than in participants with ASD. A previous study using fMRI has suggested that participants with ASD show affective empathy toward other people with ASD (15). If participants with ASD have cognitive empathy toward other people with ASD, they would show helping motivation for others with ASD. Cognitive empathy is an ability to intentionally understand other people’s emotions, while affective empathy is unintentionally sharing in other people’s emotions (40). Based on affective empathy, observing other people’s deep sadness and feeling similar sadness disturb observers’ minds. Consequently, it becomes difficult to help other people (41). On the other hand, cognitive empathy gives priority to understanding other peoples’ situation, and consequently, enables helping other people who are sad.

In the present study, participants with ASD did not show greater helping motivation for others with ASD compared to others with TD. Thus, participants with ASD did not show greater cognitive empathy even if the targets had ASD characteristics. However, individuals with ASD showed helping motivation when alexithymia and AQs were controlled. These findings suggest the possibility that adults with ASD might not notice the necessity to help people if they are not explicitly asked to assist. There is another possibility that lies in differences between ASD and TD regarding social contacts. The possibility could be that individuals with ASD feel good if people leave them alone when they are sad, while TD individuals feel better when they have social contacts, such as words of encouragement or hugs (42). Of course, there could be individual differences in this effect, such that some individuals with ASD need words of encouragement or want to be hugged and some TD individuals want to be left alone. It is suggested that these issues of individual differences in ASD and TD groups be examined in future research.

Cognitive alexithymia was associated with increased empathy for story characters with ASD. It is possible that individuals with ASD share similar difficulties regarding cognitive alexithymia with story characters with ASD, and as a result, they empathize with others similar to themselves (15, 43). Additionally, it was demonstrated that Externally Oriented Thinking was associated with decreased empathy and helping motivation for story characters with ASD. Externally oriented thinking is a cognitive style that shows preference for external behavioral information instead of internal emotional information (44). Thus, individuals with high Externally Oriented Thinking focused on behavioral information of story characters (story characters did not mention help explicitly), and they did not infer story characters’ implicit needs.

Finally, social skills were associated with decreased empathy and helping motivation for story characters with ASD, and attention to detail was associated with decreased empathy for story characters with ASD. Because higher sensory reactiveness is associated with lower social functioning (18), lower social skills due to atypical sensory input would predict limited cognitive empathy and helping motivation in ASD. The characteristic of lack of attention to detail in ASD was also caused by atypical sensory perception in ASD (45). These findings suggest that the reason why individuals with ASD are considered to have limited cognitive empathy and helping motivation could be related to alexithymia and the lack of social skills and attention to detail, which are related to atypical perception.

## General Discussion

ASD and TD groups showed greater empathetic responses and greater motivation for helping in TD stories than ASD stories. These results can be interpreted in the context of in-group/out-group biases. An in-group is a social group of which a person psychologically identifies as being a member; by contrast, an out-group is a social group with which an individual does not identify very much (46, 47). Both ASD and TD groups could interact and communicate more easily with in-group members than with out-group members (42). However, both TD individuals and individuals with ASD use the majority, or “non-autistic people,” as the implied context (with whom) and the reference group (according to whom) in the assessment of autistic traits (37). Therefore, although individuals with ASD might have an in-group/out-group bias, they use the perspective of TD people when judging empathy and helping motivation in stories. In other words, whereas TD people consider majority rules by TD people (but consider minority rules by people with ASD to a lesser extent), people with ASD try to accept majority rules by TD people, even if the rules are not rules for ASD.

The findings in the present study indicate that adults with ASD empathize with other people who have ASD and show motivation to help other people with ASD if cognitive and emotional alexithymia and AQ measures (social skill, attention switching, attention to detail, communication, and imagination) are eliminated. High AQ scores are associated with higher functioning, as it is a self-rating questionnaire and a strong sense of self is required. When participants with ASD completed assessments of autistic traits, they used the perspective of TD people (37). If alexithymia and AQ are statistically controlled, individuals with ASD may help other individuals with ASD. However, in reality, many individuals with ASD have high alexithymia and AQ scores. Thus, individuals with ASD do not often show voluntary motivation to help others.

Helping motivation can originate from other motivations besides empathy and shared affect, such as targeted helping (assistance based on a cognitive understanding of the other’s specific need) or altruistic helping (5). While altruistic helping by adults with ASD is not demonstrated in this study, they may have in fact demonstrated targeted helping toward others. Future studies should examine what kinds of helping motivation are demonstrated by adults with ASD.

Finally, it is important to consider the limitations of the present study. Firstly, the study could not recruit 21 people of the same gender per group with complete data sets, which resulted in group sizes of at least 39, to reach 78% power. Secondly, we did not ask the participants if the character/context was TD or ASD related after reading the stories. Rating of ASD stories by ASD people would be an important manipulation check, which would enhance the validity of the present study. Thirdly, because we used explicit tasks to ask for cognitive empathy and helping motivation, it is difficult to discuss the effects of social contacts and social desirability. Appropriate implicit tasks should be considered for future projects. Additionally, because participants had to interact with other people during the laboratory testing in our task, testing without further social interaction would be useful for reducing stress in ASD as well as social desirability in both groups.

## Supplementary Results

Reading times more than 2 standard deviations above the mean for each participant were excluded from the analysis. A 2 (ASD participants/TD participants) × 2 (ASD outcomes/TD outcomes) × 2 (ASD contexts/TD contexts) ANOVA on reading times was conducted. Results indicated that the interaction between participants and outcomes was significant [*F*(1, 40) = 6.45, *p* < .05, *η_p_^2^* = .14]. TD participants read TD outcomes (2,468.9 ms) faster than ASD (2,852.1 ms) outcomes [*F*(1, 19) = 13.41, *p* < .05, η*_p_^2^* = .41]. However, the ASD participants did not read ASD outcomes (2,679.6 ms) faster than TD (2,592.2 ms) outcomes [*F*(1, 19) = 2.31, *p* > .05, η*_p_^2^* = .10]. The interaction between the outcomes and the contexts was significant [*F*(1, 40) = 22.57, *p* < .05, η*_p_^2^* = .99]. These results were consistent with previous studies. Reading times for TD participants were shorter for stories about TD outcomes than stories about ASD outcomes (38). The interaction between participants and contexts was not significant [*F*(1, 40) = 0.17, *p* > .05, η*_p_^2^* = .00], and the participants × outcomes × contexts interaction was not significant [*F*(1, 40) = 0.41, *p* > .05, η*_p_^2^* = .02].

A 2 × 2 × 2 ANOVA on empathetic response ratings (**Supplementary Figure 1**) was also conducted. Results indicated that the interaction between participants and outcomes was significant [*F*(1, 40) = 8.88, *p* < .05, η*_p_^2^* = .18]. ASD participants showed greater empathetic responses toward ASD outcomes than TD participants [*F*(1, 40) = 4.06, *p* < .05, η*_p_^2^* = .09], whereas TD participants showed greater empathetic responses toward TD outcomes than ASD participants [*F*(1, 40) = 5.83, *p* < .05, η*_p_^2^* = .13]. Moreover, TD participants showed greater empathetic responses toward TD outcomes than ASD outcomes [*F*(1, 40) = 5.83, *p* < .05, η*_p_^2^* = .13]. ASD participants did not show greater empathetic responses toward ASD outcomes than TD outcomes [*F*(1, 40) = 4.06, *p* > .05, η*_p_^2^* = .09]. The interaction between participants and context was significant [*F*(1, 40) = 7.18, *p* < .05, η*_p_^2^* = .15]. TD participants showed greater empathetic responses toward the TD contexts than ASD contexts [*F*(1, 40) = 26.36, *p* < .001, η*_p_^2^* = .58].

A 2 × 2 × 2 ANOVA on motivation for helping ratings was also conducted, which indicated that the interaction between participants and outcomes was significant [*F*(1, 40) = 7.92, *p* < .05, η*_p_^2^* = .17]. **Supplementary Figure 2** shows that TD participants showed greater motivation for helping toward TD outcomes than ASD participants [*F*(1, 40) = 13.94, *p* < .05, η*_p_^2^* = .26], whereas ASD participants did not show greater motivation for helping toward ASD outcomes than TD participants [*F*(1, 40) = 0.06, *p* > .05, η*_p_^2^* = .00]. Additionally, while TD participants showed greater motivation for helping toward TD outcomes than ASD outcomes [*F*(1, 40) = 13.86, *p* < .05, η*_p_^2^* = .26], ASD participants did not show greater motivation for helping toward ASD outcomes than TD outcomes [*F*(1, 40) = 0.06, *p* > .05, η*_p_^2^* = .00]. These results indicated that the helping motivation of individuals with ASD was similar for ASD and TD targets, whereas the helping motivation in TD individuals was higher for TD than for ASD targets.

## Ethics Statement

Our protocol was in accordance with the Declaration of Helsinki and was approved by the Ethics Committee of the University of Fukui (Japan). Before participation, written informed consent was obtained from each participant. All methods were carried out in accordance with the approved protocol.

## Author Contributions

HKom, HKos, and HO developed the concept of the study. All authors contributed to the study design of the study. TF and MJ performed the data analysis and interpretation under the supervision of HKos and HO. HKom and HKos drafted the manuscript. TF, MJ, and HO provided critical revisions. All authors approved the final version of the manuscript for submission.

## Funding

This research was funded, in part, by Grant-in-Aid for Scientific Research from the Japan Society for the Promotion of Science (26118505, 15K13116, 16H01507, 16H02837, 16K17469, 18K03034) and supported by Pfizer Health Research Foundation, Aoyama Gakuin University Research Institute (research unit “Projection Science”).

## Conflicts of Interest Statement

The authors declare that the research was conducted in the absence of any commercial or financial relationships that could be construed as a potential conflict of interest.

## Footnotes

1 It is often the case in real life that ASD people behave like TD individuals when interacting with TD people (37). We therefore conducted a 2 (ASD participants/TD participants) × 2 (ASD outcomes/TD outcomes) × 2 (ASD contexts/TD contexts) ANOVA (see **Supplementary Results**).

2 The name of the protagonist was mentioned when rating empathy and helping motivation.

3 Three individuals with ASD chose not to complete questions on the AQ scale. Thus, total AQ, social skill, attention switching, attention to detail, communication, and imagination subscales were collected for the remaining 19 individuals with ASD.

## Supplementary Material

Supplementary Figure 1 The empathic responses ratings for each story of ASD (left) and TD (right) groups. 1: Least empathy - 4: Neutral- 7: Greatest empathy.

Supplementary Figure 2 The helping motivation ratings for each story of ASD (left) and TD (right) groups. 1: Least motivation - 4: Neutral- 7: Greatest motivation.
