# Supplementary figures and images for: Do Individuals With Autism Spectrum Disorders Help Other People With Autism Spectrum Disorders? An Investigation of Empathy and Helping Motivation in Adults With Autism Spectrum Disorder
Source: Front Psychiatry. 2019 Jun 4;10:376. doi: 10.3389/fpsyt.2019.00376 (PMC6558937; doi:10.3389/fpsyt.2019.00376)

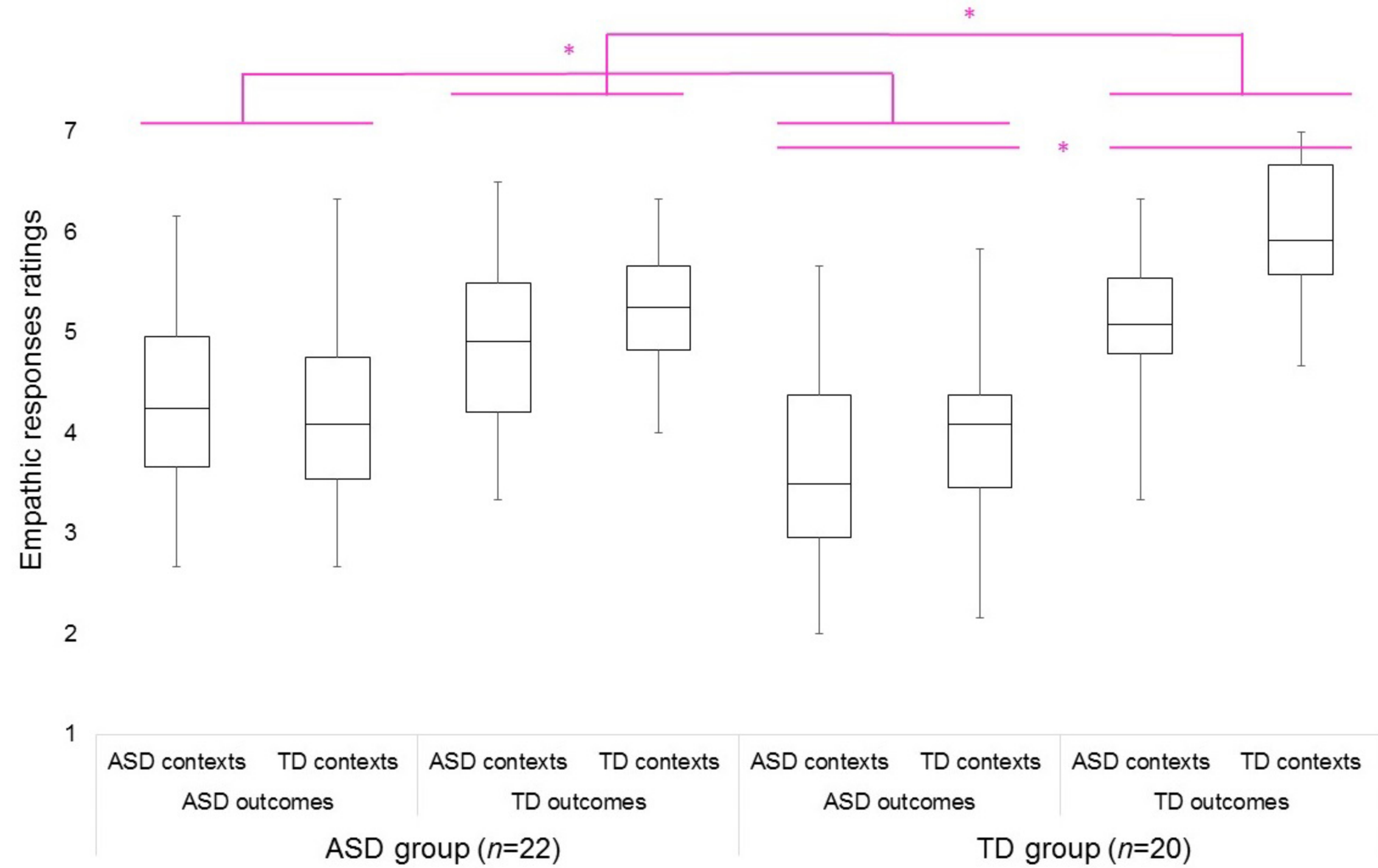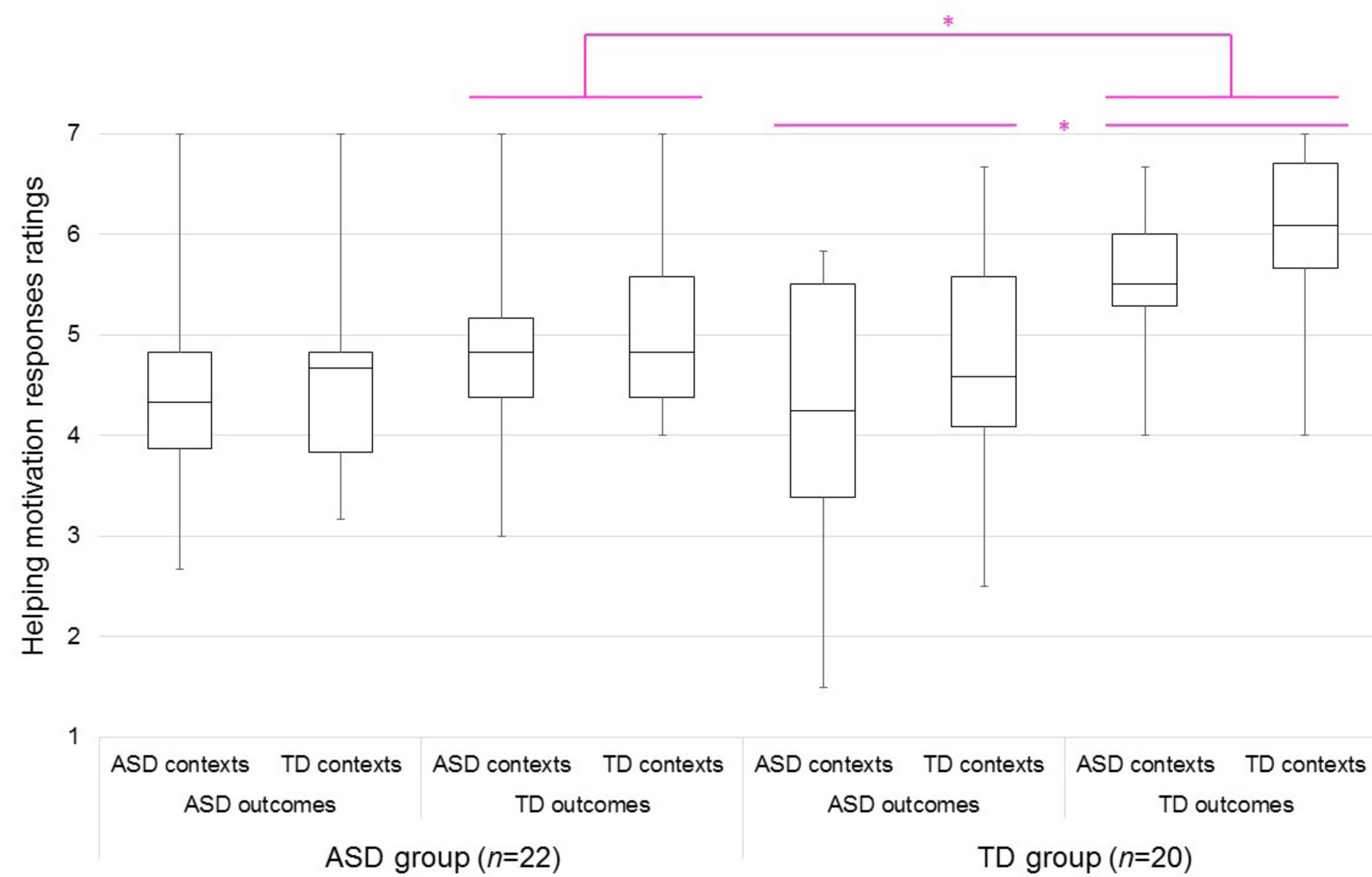

Supplement: Supplementary Figure 1 — The empathic responses ratings for each story of ASD (left) and TD (right) groups. 1: Least empathy - 4: Neutral- 7: Greatest empathy. [file DataSheet_1.pdf]
